# Complete Genome Sequence of Bacillus velezensis Strain S4, Isolated from Biochar-Treated Soil

**DOI:** 10.1128/MRA.00352-20

**Published:** 2020-05-14

**Authors:** Priscilla P. Hempel, Mengyin Yao, Sarah Yannarell, Olga Shevchenko, Franziska Vogt, Nicole Donofrio, Julia A. Maresca

**Affiliations:** a Center for Bioinformatics and Computational Biology, University of Delaware, Newark, Delaware, USA; b Department of Civil and Environmental Engineering, University of Delaware, Newark, Delaware, USA; c Department of Chemistry and Biochemistry, University of Delaware, Newark, Delaware, USA; d University of Delaware Sequencing and Genotyping Center, Newark, Delaware, USA; e Department of Plant and Soil Sciences, University of Delaware, Newark, Delaware, USA; University of Arizona

## Abstract

Here, we report the complete genome sequence of Bacillus velezensis strain S4, which was isolated from biochar-amended agricultural soil collected in Smyrna, Delaware. The genome is 4.07 Mbp, encodes 3,918 predicted proteins, and has a GC content of 46.4%.

## ANNOUNCEMENT

While investigating biochar-amended soils, we isolated a *Bacillus* strain. Since soil *Bacillus* spp. often produce antibiotics (1), we report the genome sequence of Bacillus velezensis strain S4 and its potential to synthesize antimicrobial compounds.

Biochar-amended agricultural soil (∼0.1 g) was collected in 2011 in Smyrna, Delaware, diluted 1:10^−10^ in sterile water, spread onto Luria-Bertani (LB) agar, and incubated at 28°C until colonies appeared. Individual colonies were restreaked onto LB agar until they were axenic, as determined by microscopy. Isolates were identified by Sanger sequencing of 16S amplicons (primers 8F and 1492R [2]).

Strain S4 was revived on LB agar from a −80°C stock (10% glycerol). One colony was restreaked onto LB agar and grown overnight at 28°C, and then one colony was transferred to 50 ml LB medium and grown overnight. DNA was extracted from 3.5 ml of this culture (Monarch DNA extraction kit, NEB product number T3010).

Single-molecule real-time (SMRT) libraries were prepared using the standard PacBio protocol for 20-kb libraries. DNA fragments larger than 6 kb were size selected using BluePippin (Sage Science). The average fragment size of the library was 12 kb, as measured with a fragment analyzer (Advanced Analytical Technologies, Inc.). Sequencing was completed on a PacBio RS II sequencer in one SMRT cell using P6-C4 chemistry with a 6-h movie.

This sequencing resulted in 150,292 reads (average length, 13,783 bp [range, 0 to 74,529 bp]). Reads were filtered by quality of ≥0.8 and length of ≥1 kb using the PreAssembler Filter v1 protocol within the SMRT Analysis v2.3.0 software (Pacific Biosciences) (3); 90,373 reads passed filtering (average length, 19,683 bp [range, 1,000 to 74,592 bp]), and 84,594 reads were assembled using HGAP3 (4), with 15-kb seed read fragment lengths. This assembly resulted in one contig (4,075,299 bp), which was plotted against itself using Gepard v1.4 (5) (zoom, 5,781; word length, 10; window size, 0; use matrix, DNA). The region of overlap was identified (zoom, 7; word length, 10; window size, 0; use matrix, DNA) and trimmed using custom scripts (https://github.com/MarescaLab/contig_to_linear_genome), producing a 4,065,174-bp circular chromosome with 315× coverage and a GC content of 46.4%. There is a 1-nucleotide discrepancy in the region of overlap at position 1318741. We included the extra nucleotide in the assembly because the sequence with the insertion has greater coverage (305×, compared to <30×), and the top BLASTn hit for the predicted gene (BVELS4_01369) has the additional nucleotide.

Prokka v1.11 (6) and barrnap v0.7 (https://github.com/tseemann/barrnap) were used to predict open reading frames using default parameters. This genome contains 9 rRNA operons, 86 tRNA genes, 1 transfer mRNA gene, and 3,918 predicted protein-coding genes.

Based on its 16S gene sequence, isolate S4 was initially identified as a strain of Bacillus amyloliquefaciens subsp. *plantarum,* which has been reclassified as B. velezensis subsp. *plantarum* (7, 8). Pairwise average nucleotide identity based on BLAST (ANIb) values comparing isolate S4 to 10 other *Bacillus* spp. (7) were calculated using PyANI v0.2.9 (9) with default parameters in anvi’o v6.1 (10). The genome of isolate S4 is most similar to that of B. velezensis subsp. *plantarum* FZB42 (GenBank accession number CP000560.1) (ANIb, 0.9106) and quite distant from that of Bacillus pumilus 150a (CP027034.1) (ANIb, 0.2497).

AntiSMASH v5.1.0 (11) (bacterial version, program defaults) identified nine gene clusters with >90% similarity to known antibiotic gene clusters. The predicted products include lipopeptides, polyketides, lanthipeptides, and non-ribosomally synthesized peptides.

### Data availability.

The raw reads have been deposited in the Sequence Read Archive under accession number PRJNA612570. The assembly is available at NCBI under the accession number CP050424.
